# Boost IORT in Breast Cancer: Body of Evidence

**DOI:** 10.1155/2014/472516

**Published:** 2014-09-02

**Authors:** Felix Sedlmayer, Roland Reitsamer, Christoph Fussl, Ingrid Ziegler, Franz Zehentmayr, Heinz Deutschmann, Peter Kopp, Gerd Fastner

**Affiliations:** ^1^Department of Radiotherapy and Radio-Oncology, LKH Salzburg, General Hospital, Paracelsus Medical University Clinics, Muellner Hauptstrasse 48, 5020 Salzburg, Austria; ^2^Institute of Research and Development of Advanced Radiation Technologies (radART), Paracelsus Medical University, 5020 Salzburg, Austria; ^3^Department of Special Gynecology and Breast Center, General Hospital, Paracelsus Medical University Clinics, 5020 Salzburg, Austria

## Abstract

The term IORT (intraoperative radiotherapy) is currently used for various techniques that show decisive differences in dose delivery. The largest evidence for boost IORT preceding whole breast irradiation (WBI) originates from intraoperative electron treatments with single doses around 10 Gy, providing outstandingly low local recurrence rates in any risk constellation also at long term analyses. Compared to other boost methods, an intraoperative treatment has evident advantages as follows. *Precision.* Direct visualisation of the tumour bed during surgery guarantees an accurate dose delivery. This fact has additionally gained importance in times of primary reconstruction techniques after lumpectomy to optimise cosmetic outcome. IORT is performed before breast tissue is mobilised for plastic purposes. *Cosmesis.* As a consequence of direct tissue exposure without distension by hematoma/seroma, IORT allows for small treatment volumes and complete skin sparing, both having a positive effect on late tissue tolerance and, hence, cosmetic appearance. *Patient Comfort.* Boost IORT marginally prolongs the surgical procedure, while significantly shortening postoperative radiotherapy. Its combination with a 3-week hypofractionated external beam radiotherapy to the whole breast (WBI) is presently tested in the HIOB trial (hypofractionated WBI preceded by IORT electron boost), a prospective multicenter trial of the International Society of Intraoperative Radiotherapy (ISIORT).

## 1. Introduction

Globally, breast cancer incidence rates are highest in North America and northern Europe. Breast cancer mortality rates have declined since 1975, attributed to the increased use of screening mammography and greater use of adjuvant treatments, including radiotherapy. For locoregional treatment, breast-conserving therapy is regarded as standard of care, comprising breast conserving surgery followed by ipsilateral whole-breast radiotherapy (WBRT) as an integral component.

Postoperative radiotherapy significantly reduces local recurrence rates. The more pronounced the achieved reduction, the more substantially it translates into improved survival. Four prevented local recurrences result in one avoided breast cancer death [1, 2].

## 2. Rationale for IORT and Biology of Single High Doses

Pathological analysis revealed that the greatest tumour cell density (up to 90% of microscopic remainders) is observed in an area of 4 cm surrounding the macroscopic tumour edge [3, 4]. As a consequence, after a breast-conserving operation, the tumour bed represents the region with the highest probability of in-breast recurrence. Therefore, an additional booster dose to the tumour bed significantly reduces local recurrence rates [5]. Up to now, these boosts are traditionally still applied mostly by external beam electrons of 10–16 Gy (5–8 × 2 Gy) or, alternatively, by interstitial implants (HDR-brachytherapy).

The idea of intraoperative irradiation (IORT) during breast-conserving surgery is the delivery of a high single boost dose to the area at highest risk for subclinical tumour cell contamination with utmost precision, due to direct visualisation. The method was originally introduced by the Medical College of Ohio (MCO) in Toledo, Ohio, USA, and the Centre Regional de Lutte Contre Le Cancer (CRLC) in Montpellier, France, based on reports of 72 patients treated with an electron boost (intraoperative electron radiotherapy (IOERT)) [6–8].

Compared to squamous cell carcinoma, breast cancer seems to show a different sensitivity towards higher single doses. In 1989, Fowler postulated an alpha/beta ratio of 4 for breast cancer as its best approximation instead of 10 for most SCC [9]. This value was strongly supported by the clinical outcome of Canadian and British Hypofractionation Trials [10, 11]. A lower ratio results in higher sensitivity against higher doses per fraction, an argument clearly in favour of IORT. In the linear quadratic model, using an alpha/beta value of 4, an IORT dose of 10 Gy amounts to a BED of 35, hence, being isoeffective to a boost of about 24 Gy when applied in single fractional doses of 2 Gy. However, the model was only tested for single doses below 15 Gy [12]. The prediction of isoeffects of doses above this level leaves open questions and has to be further evaluated.

Beyond these dose-effect extrapolations, it was hypothesized that immediate irradiation during surgery has implications on the tumor microenvironment abrogating the proliferative cascade induced by surgical wound healing. In vitro, wound fluid has been described to stimulate tumor cell proliferation and invasion, which can be blocked by a high-dose IORT [13]. Another aspect is the prevention of possible residual tumor cell repopulation between surgery and adjuvant radiotherapy. Furthermore, a good oxygenation status of the tumor bed during operation could also be a factor for enhanced biological effectiveness, which has not been investigated yet. All these cellular and transcellular reactions of irradiated tissues are neither clarified in detail nor understood in their particular impact on clonogenic cell inactivation—and hence, local control—and are subject of ongoing research [14–16].

## 3. IORT Treatment Methods

In the late 1990s, a broad clinical IOERT application started at the European Institute of Oncology (EIO) in Milan, Italy, and the Paracelsus Medical University (PMU) in Salzburg, Austria. Since then, IORT to the tumour bed during breast-conserving surgery has become a booming field of interest for partial breast irradiation, either as an anticipated boost or as the sole treatment strategy in limited-stage breast cancer.

This has given rise to the development of different technical approaches, with the term “IORT” now primarily used for the following techniques: low-kV orthovolt systems (e.g., Intrabeam) and intraoperative radiotherapy with electrons on mobile or standard linear accelerators (IOERT).

The orthovolt systems consist of a miniature, electron driven, low-kV energy X-ray source, emitting an isotropic X-ray spectrum. For breast irradiations, spheric applicators, chosen according to the excision cavity's size, are put at the top of the source, resulting in a spherical dose delivery with very steep dose fall-off.

*Linac-Based IOERT* is possible with various electron energies (4–18 MeV). After tumour removal, the tissue surrounding the excision cavity is surgically mobilised and temporarily approximated by sutures in order to bring adjacent walls into reach of the electron beam. The tissue thickness is usually measured by intraoperative ultrasound for individual depth dose prescription, choice of proper electron energies, and electron tube diameters providing safe coverage of the tumour bed, respectively, building clear dosimetric and volumetric advantages in comparison to the kV system.

The dosimetric properties of the two IORT methods in terms of dose homogeneity, flexibility towards asymmetric target volume shapes, and their consequent ability to deliver a reliable dose to a given volume differ tremendously [17]. Outcome analyses of local control rates as well as cosmetic results after “IORT” must strictly be performed according to the used technique.

## 4. IORT Treatment Concepts: Boost versus Single Modality

For both technical IORT approaches (electrons and orthovolt), two different treatment concepts are proposed:

- IORT as full dose partial breast irradiation (PBI) with either electrons (ELIOT) or orthovolt devices (TARGIT);
- IORT as anticipated boost followed by whole-breast radiotherapy (WBI) (BIO-boost = breast intraoperative boost).

Pros and cons of PBI strategies including application by IORT are matter of ongoing debate [18, 19]. Interpretation of the so-far results following sole IORT—by any means—to be isoeffective towards whole-breast treatment strategies is premature, the respective trials show partially conflicting results and, most important, median follow-up periods below 4 years [20, 21]. True local recurrences, however, are presumed to occur between 40 and 65 months after primary treatment [22, 23], out-quadrant relapses even later than that [24] when WBI was performed. Only adequate long term experience will reveal the potential of a sole IORT approach to replace WBI in selected patient groups, which is not yet the case.

Therefore, this paper focuses on results after Boost IORT, where the body of evidence shows by far higher maturity.

Boost IORT addresses the question of whether this approach is an effective and/or superior alternative to conventional boost techniques. The advocates of a BIO-boost emphasise the use of lower single doses compared with a full-dose concept, with dose ranges well understood in terms of tumour effects and late tissue reactions. Since IORT is followed by WBI, the concept still accounts for the (unknown) risk of occult tumour burden in distant quadrants. Therefore, it is less vulnerable to possible underdosage in the periphery of a tumour bed and—unlike PBI concepts—remains applicable in every risk constellation.

## 5. Clinical Results

### 5.1. Boost IORT with Electrons (IOERT)

Despite its retrospective character, the International Society of Intraoperative Radiotherapy (ISIORT) Europe pooled analysis on IOERT provides the best available evidence so far [25, 26].

#### 5.1.1. The ISIORT Europe Pooled Analysis (BIO-Boost) [26]

The joint investigation evaluated the long-term outcome of the IOERT strategy aimed at reducing local recurrence in breast cancer and was carried out in a joint effort by seven institutions from Austria, Germany, Italy, and France—all members of ISIORT's European Group (ISIORT Europe). Until October 2005, 1109 unselected patients of any risk group have been identified among seven centers using identical methods, sequencing, and dosage for intra- and postoperative radiotherapy. A median IOERT dose of 10 Gy was applied (90% reference isodose), preceding WBI with 50–54 Gy (single doses 1.7–2 Gy). 60% of all patients (*n* = 655) presented with at least one of the following adverse prognostic factors for local recurrence: tumour size > 2 cm, high grade, age < 45 years, and positive lymph nodes. In the most recent long-term analysis, at a median follow-up of 72.4 months (0.8–239), only 16 in-breast recurrences were observed, half of them accounting for true local recurrences; this yields an in-breast tumour control rate of 99.2% at 73.3 months. Relapses occurred 12.5–151 months after primary treatment. In a multivariate analysis, grade 3 tumour was found to be predictive of recurrence (*P* = 0.024). A significant univariate trend was found for in-breast relapse in case of negative hormonal status and young age (below 40 years). Annual in-breast recurrence rates amounted to 0.64%, 0.34%, 0.21%, and 0.16% in patients <40 y; 40–49 y; 50–59 y, and ≥60 y, respectively. In all risk subgroups, a 10 Gy IOERT boost prior to WBI provided local control rates which compare favourably to the reported figures in all trials with similar length of follow-up, irrespective of the used boost methods [5, 27–29].

Furthermore, in a retrospective matched-pair analysis, 188 patients with external e-boost (6 × 2 Gy Dmax (1.8 Gy ref D)) were compared to the pooled analysis' first 190 patients from Salzburg IOERT (10 Gy Dmax (9 Gy ref D)) [30]. At 10-year follow-up, the in-breast recurrence rate in the external e-boost group was 7.2% and 1.6% in the IOERT group, respectively. This significant difference was almost entirely due to a reduction in true local recurrence.

### 5.2. Boost IORT with Low-Kilovoltage X-Rays

Compared to IOERT, clinical long term experience following boost IORT with 50 kV X-rays is less solid, with updates of two larger cohorts available [31, 32].

In a multicenter pilot study prior to the international Targit A-trial, the feasibility of 50 kV-IORT was tested among participating institutions [31]. Between 1998 and 2005, 299 patients were treated with a 20 Gy IORT boost (surface dose) during breast conserving surgery (BCS), followed by 45–50 Gy WBI (2 Gy single fraction) after wound healing or completion of adjuvant chemotherapy when indicated. After a median follow-up of 5 years, eight ipsilateral tumor recurrences were reported, resulting in a recurrence rate estimate of 1.73% (SE 0.77, Kaplan-Meier) and an observed rate of 2.67%, respectively. The authors concluded that, for patients with similar selection at risk for in-breast relapse, their findings compared favorably to the tumor control rates achieved in the EORTC boost trial [5] or the START-B trial [29].

A second series provides a single-center experience following kV-boosts with 18–20 Gy plus WBI (46–50 Gy/2 Gy per fraction) [32]. A total of 197 patients were treated, with the cohorts partially overlapping with the multicenter study. The last update was published at a median follow-up period of 37 months. At this point of time, six in-breast events were recorded (5 invasive, one in situ relapse), accounting for a 3% in-breast recurrence rate (both at 3 and at 5 years).

Compared to the results following IOERT, these rates look somewhat inferior, despite similar patient selection. In addition, sample sizes were significantly lower, and follow-up was also less mature. However, a definitive answer on the superiority of a respective IORT method would necessitate a randomized trial. Indirect evidence on the basis of observational head-to-head comparisons indicates an obvious trend in favor of IOERT. A possible explanation might be the superior coverage of breast tissue by electrons, where larger volumes of a putative tumor bed are treated with uniformly high doses. Moreover, clinical target volumes often appear asymmetric, which is better compensated by IOERT in terms of spatial direction of dose deposition (margin-directed applicator guidance) [17].

### 5.3. Boost IOERT after Primary Systemic (Neoadjuvant) Treatment

In patients with locally advanced breast cancer (LABC), in-breast tumor recurrence rates (IBTR) are reported to be increased after breast BCS following primary systemic treatment (PST). To date, there are no publications on the effect of an IOERT boost in these high-risk constellations. In a retrospective analysis, a subgroup from the Salzburg cohort was evaluated for subsequent IBTR following PST, BCS, and IOERT preceding whole breast irradiation [33].

Eighty-three patients with clinical stage II or III breast cancer treated between 2002 and 2007 were identified and analysed in 2012. Patients received 3 to 6 cycles of anthracycline/taxane containing preoperative chemotherapy. All patients had breast conserving surgery with sentinel node biopsy and axillary lymph node dissection and received IOERT with 9 Gy to the 90% reference isodose as anticipated tumor bed boost. WBI was performed after surgery for all patients with single fractional doses of 1.7–1.8 Gy (5x/week) to total doses of 51–57 Gy.

Two patients refused WBI, leaving 81 patients for further analysis. Pathologic complete response was achieved in 14/81 (17%) patients. After a median follow-up of 59 months (range 3–115), two IBTR, both in the former index quadrant, were observed, corresponding to a local control rate (LC) of 98.5%. In this retrospective analysis, boost IOERT turned out to be a highly effective tool for the prevention of IBTR in LABC following neoadjuvant chemotherapy, likewise to its demonstrated potential in multimodal adjuvant regimen.

### 5.4. Toxicity and Cosmesis following Boost IORT

In all studies, IORT manoeuvres turned out to be safe and feasible, showing no treatment related mortality or excess acute local morbidity in terms of delayed wound healing or infection rates compared to conventional treatment [6, 34–37]. As to late reactions, cumulative incidences of fibrosis/sclerosis within the IORT volumes were slightly different according to the treatment concept: for boost patients, tolerance was excellent with incidences of 20–25% G1-2 and less than 2% G3-reactions [6, 8, 37, 38]. Following full-dose IORT, reported rates amounted up to 80% G1, 30% G2, and up to 6% G3-sequelae [39–41].

Cosmetic outcome after IORT boosts was analysed in basically three reports altogether comprising 84–122 patients. In two trials, no difference was described for the boost patients in comparison to conventional groups: results were rated as 86/91% to be good or excellent for IORT-Boosts and 81/96% for the control groups, respectively [35, 37]. Longest-term experience is provided by Lemanski et al. [8] who reported about late reactions in 42 recurrence-free patients after a median follow-up of 9 years. Six patients (14%) experienced Grade 2 late subcutaneous fibrosis within the boost area. Overall cosmesis was scored to be good to excellent. Based on their experience in 48 patients, Wenz et al. described inferior cosmetic results when time intervals between orthovoltage IORT and onset of WBI fall below 30 days [42].

In all these studies, the authors used different standardized cosmetic scoring systems based on qualitative estimations. However, in comparison to conventional techniques, no negative impacts on cosmesis following IORT have been reported so far in any concept.

#### 5.4.1. Salzburg Experience

To assess cosmetic long term results following boost IOERT with 10 Gy preceding WBI, 403 patients were evaluated by photo-documentation in four standardized positions in order to provide reproducible examination conditions [43]. Cosmetic outcome was assessed separately by patients and treating physicians (surgeon and radiation oncologist), respectively, within a 5-point-score as described by Harris and van Limbergen. Patient-, tumor- and treatment-related factors were analysed with regard to possible impact on the cosmetic result. For the whole cohort, median time between end of radiotherapy and cosmetic evaluation was 45 months; a separate subgroup analysis was carried out for 261 patients with a minimum follow-up of three years (median 56 months). For the whole cohort, the patients' self-assessments yielded around 93% “Satisfactory” (Excellent/Good) and 98% “Acceptable” (Excellent/Good/Moderate). These figures were almost identical to the scorings in the subgroup with longer follow up: 91% satisfactory and 97% acceptable scorings, respectively. Physicians' evaluation revealed 64% satisfactory and 95% acceptable results for both groups. Telangiectasia were not described at all. A significant positive correlation was found between physicians' and patients' evaluation. Age and applicator diameter (possibly as surrogate for length of surgical scar) had a significant negative impact on the cosmetic outcome, whereas, for example, tumor-stage, grading, electron-energy, and boost-volume had no significant influence. IORT as a 10 Gy electron-boost within breast conserving treatment shows in the longer follow-up excellent cosmetic results. A negative impact of factors attributable to IORT on the cosmetic outcome was not identified.

### 5.5. HIOB Trial

Hypofractionated Whole-Breast Irradiation following Intra-Operative Electron Boost. (http://www.clinicaltrials.gov/ct2/show/NCT01343459?term=hiob&rank=1).

In an attempt to further reduce overall treatment duration without compromising local control rates, the multicentre HIOB trial was started in January 2011 as an ISIORT investigator initiated study. In this trial, Boost IOERT of 10 Gy is combined with hypofractionated WBI (15 × 2.7 Gy) for stage I/II breast cancer. A similar concept of IOERT plus short-term WBRT was tested in a phase II design by the Milano Group [38]. The HIOB trial design follows a sequential probability ratio test (SPRT), defining annual in-breast recurrence rates as benchmarks for successful treatment. Superiority of the intervention is defined by falling below the best published evidence in non-IORT cohorts. Beside tumor-related endpoints, major emphasis is made on cosmetic outcome, where a first analysis on early results is available [44].

As of November 2013, within seven active institutions, 426 patients have been recruited, 336 of them already in follow-up. Perioperatively, no major complications were observed. Four weeks after the end of WBI and 336 evaluated patients, 108 (32%) showed no reactions (CTC 0), 208 (61.5%) presented with faint (CTC 1), and 20 (6%) with moderate to brisk erythema (CTC 2), respectively. G0-I late reactions (LENT-SOMA) occurred in a mean frequency of 93%, 98%, and 99% after 4-5 months and 1- and 2-year follow-up, respectively.

Cosmesis was repeatedly assessed and referenced against the baseline appearance prior to WBI, which was scored as sufficient in 85%/69% of 352 subjective/301 objective evaluations. Respective results at 4-5 months, one and two years post RT were 86%/71% of 279/227, 88%/77% of 173/75, and 88%/74% of 41/31 ratings.

At a median follow-up period of 13 months (range 1–24), two patients were metastasized, no recurrence was noted.

In sum, tolerance of a combined IOERT/hypofractionated WBI regimen was excellent, acute reactions moderate, and late reactions insignificant in short-term assessment. With regard to postoperative appearance, early cosmetic results are not impaired by this regimen. Both tumor control and cosmetic outcome have to be evaluated on long-term follow-up.

## References

[1] McGale P, Taylor C, Correa C (2014). Effect of radiotherapy after mastectomy and axillary surgery on 10-year recurrence and 20-year breast cancer mortality: meta-analysis of individual patient data for 8135 women in 22 randomised trials. *The Lancet*.

[2] Darby S, McGale P, Correa C (2011). Effect of radiotherapy after breast-conserving surgery on 10-year recurrence and 15-year breast cancer death: meta-analysis of individual patient data for 10 801 women in 17 randomised trials. *The Lancet*.

[3] Holland R, Veling SHJ, Mravunac M, Hendriks JHCL (1985). Histologic multifocality of Tis, T1-2 breast carcinomas: Implications for clinical trials of breast-conserving surgery. *Cancer*.

[4] Faverly DR, Hendriks JH, Holland R (2001). Breast carcinomas of limited extent: frequency, radiologic-pathologic characteristics, and surgical margin requirements. *Cancer*.

[5] Antonini N, Jones H, Horiot JC (2007). Effect of age and radiation dose on local control after breast conserving treatment: EORTC trial 22881–10882. *Radiotherapy and Oncology*.

[6] Merrick HW, Battle JA, Padgett BJ, Dobelbower RR (1997). IORT for early breast cancer: a report on long-term results. *Frontiers of Radiation Therapy and Oncology*.

[7] Battle JA, DuBois JB, Merrick HW, Dobelbower RR, Gunderson LL, Willett CG, Harrison LB, Calvo FA (1999). IORT for breast cancer. *Current Clinical Oncology: Intraoperative Irradiation Techniques and Results*.

[8] Lemanski C, Azria D, Thezenas S (2006). Intraoperative radiotherapy given as a boost for early breast cancer: long-term clinical and cosmetic results. *International Journal of Radiation Oncology Biology Physics*.

[9] Fowler JF (1989). The linear-quadratic formula and progress in fractionated radiotherapy. *British Journal of Radiology*.

[10] Bentzen SM, Agrawal RK, Aird EG (2008). The UK Standardisation of Breast Radiotherapy (START) Trial B of radiotherapy hypofractionation for treatment of early breast cancer: a randomised trial. *Lancet*.

[11] Whelan TJ, Kim D-H, Sussman J (2008). Clinical experience using hypofractionated radiation schedules in breast
cancer. *Seminars in Radiation Oncology*.

[12] Bartelink H (2001). Commentary on the paper “a preliminary report of intraoperative radiotherapy (IORT) in limited-stage breast cancers that are conservatively treated”: a critical review of an innovative approach. *European Journal of Cancer*.

[13] Belletti B, Vaidya JS, D'Andrea S (2008). Targeted intraoperative radiotherapy impairs the stimulation of breast cancer cell proliferation and invasion caused by surgical wounding. *Clinical Cancer Research*.

[14] Vaidya JS, Baldassarre G, Massarut S (2009). Beneficial effects of intraoperative radiotherapy on tumor microenvironment could improve outcomes. *International Journal of Radiation Oncology Biology Physics*.

[15] Herskind C, Wenz F (2009). Is there more to intraoperative radiotherapy than physical dose?. *International Journal of Radiation Oncology Biology Physics*.

[16] Herskind C, Wenz F (2010). Radiobiological comparison of hypofractionated accelerated partial-breast irradiation (APBI) and single-dose intraoperative radiotherapy (IORT) with 50-kV X-rays. *Strahlentherapie und Onkologie*.

[17] Nairz O, Deutschmann H, Kopp M (2006). A dosimetric comparison of IORT techniques in limited-stage breast cancer. *Strahlentherapie und Onkologie*.

[18] Sautter-Bihl M-L, Sedlmayer F, Budach W (2010). Intraoperative radiotherapy as accelerated partial breast irradiation for early breast cancer: beware of one-stop shops?. *Strahlentherapie und Onkologie*.

[19] Bartelink H, Bourgier C, Elkhuizen P (2012). Has partial breast irradiation by IORT or brachytherapy been prematurely introduced into the clinic?. *Radiotherapy and Oncology*.

[20] Veronesi U, Orecchia R, Maisonneuve P (2013). Intraoperative radiotherapy versus external radiotherapy for early breast cancer (ELIOT): a randomised controlled equivalence trial. *The Lancet Oncology*.

[21] Vaidya JS, Wenz F, Bulsara M (2014). Risk-adapted targeted intraoperative radiotherapy versus whole-breast radiotherapy for breast cancer: 5-year results for local control and overall survival from the TARGIT—a randomised trial. *The Lancet*.

[22] Gujral DM, Sumo G, Owen JR (2011). Ipsilateral breast tumor relapse: local recurrence versus new primary tumor and the effect of whole-breast radiotherapy on the rate of new primaries. *International Journal of Radiation Oncology, Biology, Physics*.

[23] Brooks JP, Danforth DN, Albert P (2005). Early ipsilateral breast tumor recurrences after breast conservation affect survival: an analysis of the National Cancer Institute randomized trial. *International Journal of Radiation Oncology Biology Physics*.

[24] Freedman GM, Anderson PR, Hanlon AL, Eisenberg DF, Nicolaou N (2005). Pattern of local recurrence after conservative surgery and whole-breast irradiation. *International Journal of Radiation Oncology Biology Physics*.

[25] Sedlmayer F, Fastner G, Merz F (2007). IORT with electrons as boost strategy during breast conserving therapy in limited stage breast cancer: results of an ISIORT pooled analysis. *Strahlentherapie und Onkologie*.

[26] Fastner G, Sedlmayer F, Merz F (2013). IORT with electrons as boost strategy during breast conserving therapy in limited stage breast cancer: long term results of an ISIORT pooled analysis. *Radiotherapy and Oncology*.

[27] Bartelink H, Horiot J-C, Poortmans PM (2007). Impact of a higher radiation dose on local control and survival in breast-conserving therapy of early breast cancer: 10-year results of the randomized boost versus no boost EORTC 22881–10882 trial. *Journal of Clinical Oncology*.

[28] Clark M, Collins R, Darby S (2005). Effects of radiotherapy and of differences in the extent of surgery for early breast cancer on local recurrence and 15 year survival: an overview of randomised trials. Early Breast Cancer Trialists' Collaborative Group (EBCTCG). *The Lancet*.

[29] Haviland JS, Owen JR, Dewar JA (2013). The UK standardisation of breast radiotherapy (START) trials of radiotherapy hypofractionation for treatment of early breast cancer: 10-year follow-up results of two randomised controlled trials. *The Lancet Oncology*.

[30] Fastner G, Reitsamer R, Kopp M (2011). Intraoperative (IOERT) versus external electron boost in breast conserving-operated breast cancer patients. 10-year results of a matched-pair analysis. *Strahlentherapie und Onkologie*.

[31] Vaidya JS, Baum M, Tobias JS (2011). Long-term results of TARGeted intraoperative radioTherapy (Targit) boost during breast-conserving surgery. *International Journal of Radiation Oncology Biology Physics*.

[32] Blank E, Kraus-Tiefenbacher U, Welzel G (2010). Single-center long-term follow-up after intraoperative radiotherapy as a boost during breast-conserving surgery using low-kilovoltage x-rays. *Annals of Surgical Oncology*.

[33] Fastner G, Reitsamer R, Ziegler I (2014). IOERT as anticipated tumor bed boost during breast-conserving surgery after neoadjuvant chemotherapy in locally advanced breast cancer—results of a case series after 5-year follow-up. *International Journal of Cancer*.

[34] Vaidya JS, Baum M, Tobias JS (2001). Targeted intra-operative radiotherapy (Targit): an innovative method of treatment for early breast cancer. *Annals of Oncology*.

[35] Ciabattoni A, Fortuna G, Ciccone V (2004). IORT in breast cancer as boost: preliminary results of a pilot randomized study on use of IORT for Stage I and II breast cancer [Abstract]. *Radiotherapy & Oncology*.

[36] Reitsamer R, Peintinger F, Sedlmayer F (2002). Intraoperative radiotherapy given as a boost after breast-conserving surgery in breast cancer patients. *European Journal of Cancer*.

[37] Kraus-Tiefenbacher U, Bauer L, Kehrer T, Hermann B, Melchert F, Wenz F (2006). Intraoperative radiotherapy (IORT) as a boost in patients with early-stage breast cancer—acute toxicity. *Onkologie*.

[38] Ivaldi GB, Leonardi MC, Orecchia R (2008). Preliminary results of electron intraoperative therapy boost and hypofractionated external beam radiotherapy after breast-conserving surgery in premenopausal women. *International Journal of Radiation Oncology Biology Physics*.

[39] Mussari S, Della Sala WS, Busana L (2006). Full-dose intraoperative radiotherapy with electrons in breast cancer: first report on late toxicity and cosmetic results from a single-institution experience. *Strahlentherapie und Onkologie*.

[40] Ollila DW, Klauber-DeMore N, Tesche LJ (2007). Feasibility of breast preserving therapy with single fraction in situ radiotherapy delivered intraoperatively. *Annals of Surgical Oncology*.

[41] Lemanski C, Azria D, Gourgou-Bourgade S (2013). Electrons for intraoperative radiotherapy in selected breast-cancer patients: late results of the Montpellier phase II trial. *Radiation Oncology*.

[42] Wenz F, Welzel G, Keller A (2008). Early initiation of external beam radiotherapy (EBRT) may increase the risk of long-term toxicity in patients undergoing intraoperative radiotherapy (IORT) as a boost for breast cancer. *Breast*.

[43] Fussl C, Merz F, Fussl A (2012). Evaluation of cosmetic Long-term results in early breast cancer after intraoperative Radiotherapy (IORT) as part of breast-conserving Therapy [Abstract]. *Strahlentherapie und Onkologie*.

[44] Fastner G, Reitsamer R, Kopp P (2014). Hypofractionated WBI plus IOERT-boost in early stage breast cancer (HIOB): updated results of a prospective trial. *Radiotherapy & Oncology*.

